# High-flow nasal cannula *versus* nasal cannula for advanced bronchoscopy

**DOI:** 10.1183/23120541.01347-2025

**Published:** 2026-03-09

**Authors:** Regina Pikman Gavriely, Ophir Freund, Amir Bar-Shai, Evgeni Gershman

**Affiliations:** 1 Faculty of Medicine, Tel Aviv University, Tel Aviv, Israel; 2 Institute of Pulmonary Medicine, Tel Aviv Sourasky Medical Center, Tel Aviv, Israel

## Abstract

We read with interest the correspondence of our colleagues to our article titled “Laryngeal mask airway or high-flow nasal cannula *versus* nasal cannula for advanced bronchoscopy: a randomised controlled trial” [1], and would like to respond to their remarks.

## Shareable abstract

**Providing some answers that explain aspects questioned by colleagues, such as preoxygenation and intervention protocols, and their influence on our results** https://bit.ly/4qBr4X1

*Reply to Y. Cao:*

We read with interest the correspondence of our colleagues to our article titled “Laryngeal mask airway or high-flow nasal cannula *versus* nasal cannula for advanced bronchoscopy: a randomised controlled trial” [1], and would like to respond to their remarks.

Firstly, the authors are right that pre-oxygenation was different than normally used in real-world practice with high-flow nasal cannula (HFNC). This was done to enable blind allocation, as patients were unaware of the method of oxygenation. We agree that the implementation of HFNC may indeed lengthen the apnoeic time and further delay or prevent hypoxia during the procedure due to the de-nitrogenation effect and oxygen reservoir expansion of HFNC. Compared to the studies cited in our paper and by the authors [2, 3], which included only endo-bronchial ultrasound, almost half of our cases had additional bronchoscopic procedures, which could possibly explain the higher rates of desaturation. We also used an approximate flow of 45 L·min^−1^, while in the other two trials higher oxygen flows were utilised. It is important to point out that considering the sample size in our study and those mentioned, the 26% rate of desaturation in our trial is not statistically different from the 10–13% in others. Of note, despite the lack of HFNC pre-oxygenation in our study, the HFNC group had the lowest rate of desaturation, an advantage that indeed can be magnified by extending its use to the pre-oxygenation period.

Secondly, the authors mention the higher levels of CO_2_ found in the HFNC group compared to the nasal cannula or laryngeal mask groups. In our cohort, respiratory interventions in the nasal cannula group mainly included oxygen enrichment *via* the bronchoscope port, chin lift and jaw thrust (leading to reopening of airway and return to spontaneous ventilation). In six cases where these methods did not provide a solution for the desaturation, temporary mask ventilation was provided, and three of these patients were ultimately switched to laryngeal mask airway support. When used, masked ventilation was applied for less than one minute, hence any effect on overall CO_2_ levels might not be significant. Still, we agree with the authors that these actions might have affected our results, given that we performed an intention-to-treat analysis. Other than the already-mentioned possible mechanisms for this trend in our discussion, the difference between procedural and post-procedural CO_2_ levels in the HFNC groups might be explained by the mouth position. Per protocol in both HFNC and NC groups bronchoscopy was done *via* a bite block mouth piece, thus keeping the mouth open. The open mouth position during bronchoscopy could lower the pressure generated by the HFNC by over 50% [4, 5], which is not the case after procedure cessation when the bite block it taken out and the mouth is closed. While the peak levels of CO_2_ were statistically higher in the HFNC during the procedure, this might not be clinically significant (mean 65.6 *versus* 60.1 mmHg), moreover there was no difference in the change of CO_2_ level from baseline in between groups.

We hope that these explanations provide more insight into our work and urge other studies in this field in order to improve the respiratory support methods for bronchoscopic examinations.
